# Editor’s Highlight: Complete Attenuation of Mouse Lung Cell Proliferation and Tumorigenicity in CYP2F2 Knockout and CYP2F1 Humanized Mice Exposed to Inhaled Styrene for up to 2 Years Supports a Lack of Human Relevance

**DOI:** 10.1093/toxsci/kfx141

**Published:** 2017-07-13

**Authors:** George Cruzan, James S. Bus, Marcy I. Banton, Satinder S. Sarang, Robbie Waites, Debra B. Layko, James Raymond, Darol Dodd, Melvin E. Andersen

**Affiliations:** * ToxWorks, Bridgeton, New Jersey 08302; † Exponent, Inc., Midland, Michigan 48640; ‡ Lyondell Chemical Company, Houston, Texas 77010; § Shell International, Houston, Texas 77002; ¶ SABIC Innovative Plastics US LLC, Mount Vernon, Indiana 47620; ‖ Hamner, Research Triangle Park, North Carolina 27709; ‖| Charles River Laboratories, Inc., Frederick, Maryland 21701; ‖‖ Charles River, Spencerville, Ohio 45887;; # ScitoVation LLC, Research Triangle Park, North Carolina 27709-5566

**Keywords:** styrene, CYP2F2-knockout mice, CYP2F1 humanized mice, proliferative lesions, chronic study

## Abstract

Styrene is a mouse-specific lung carcinogen, and short-term mode of action studies have demonstrated that cytotoxicity and/or cell proliferation, and genomic changes are dependent on CYP2F2 metabolism. The current study examined histopathology, cell proliferation, and genomic changes in CD-1, C57BL/6 (WT), CYP2F2(−/−) (KO), and CYP2F2(−/−) (CYP2F1, 2B6, 2A13-transgene) (TG; humanized) mice following exposure for up to 104 weeks to 0- or 120-ppm styrene vapor. Five mice per treatment group were sacrificed at 1, 26, 52, and 78 weeks. Additional 50 mice per treatment group were followed until death or 104 weeks of exposure. Cytotoxicity was present in the terminal bronchioles of some CD-1 and WT mice exposed to styrene, but not in KO or TG mice. Hyperplasia in the terminal bronchioles was present in CD-1 and WT mice exposed to styrene, but not in KO or TG mice. Increased cell proliferation, measured by KI-67 staining, occurred in CD-1 and WT mice exposed to styrene for 1 week, but not after 26, 52, or 78 weeks, nor in KO or TG mice. Styrene increased the incidence of bronchioloalveolar adenomas and carcinomas in CD-1 mice. No increase in lung tumors was found in WT despite clear evidence of lung toxicity, or, KO or TG mice. The absence of preneoplastic lesions and tumorigenicity in KO and TG mice indicates that mouse-specific CYP2F2 metabolism is responsible for both the short-term and chronic toxicity and tumorigenicity of styrene, and activation of styrene by CYP2F2 is a rodent MOA that is neither quantitatively or qualitatively relevant to humans.

Styrene (CAS No. 100-41-4) is used to manufacture a wide range of commercial and household products made from polystyrene, acrylonitrile-butadiene-styrene (ABS), styrene-butadiene rubber (SBR), latexes, and glass fiber-reinforced composites (boats, tub/showers). Styrene has been extensively studied for a variety of human health and environmental effects.

A key health concern is the potential human relevance of lung toxicity and tumorigenicity observed in multiple mouse but not rat studies (Cruzan *etal.*, 1998, 2001). An understanding of the human relevance of mouse lung tumors is particularly important in that these findings are observed at styrene exposures as low as 20 ppm (Cruzan *etal.*, 2001), and similar exposures are encountered in some occupational scenarios such as fiberglass composite workers (Collins *etal.*, 2013; Triebig *etal.*, 2009). However, short-term mode of action studies have strongly supported the hypothesis that lung toxicity and tumorigenicity observed only in mice is attributable to mouse-lung specific metabolism of styrene to ring-oxidized metabolite(s) by mouse CYP2F2 (Cruzan *etal.*, 2002, 2005, 2009, 2012, 2013). The most robust evidence supporting this hypothesis is found in short-term oral or intraperitoneal dose mode of action studies in which the lung toxicity of styrene and a previously postulated putative lung toxic metabolite, styrene oxide (a primary metabolite of CYP2E1 metabolism; Green *etal.*, 2001), was completely attenuated in CYP2F2 knockout mice (Cruzan *etal.*, 2012) and in mice in which human CYP2F1 was inserted as a transgene in the knockout mouse strain (Cruzan *etal.*, 2013).

Despite robust mode of action data indicating the essential role of styrene CYP2F2 metabolism in mediating the lung toxicity of styrene, these data do not fully address the possibility that alternative and potentially human-relevant mode(s) of action outside of CYP2F2 metabolism, eg, styrene oxide formation by CYP2E1, might be contributing to styrene-induced mouse lung tumors. It is important to note, however, that the styrene oxide hypothesis is inconsistent with the observation that styrene is not a lung carcinogen in rats even after 2 years of 6 h/day, 5 days/week exposures up to 1000 ppm styrene, with plasma styrene oxide concentrations substantially higher in rats at this dose compared with mice exhibiting tumors at substantially lower exposures (Cruzan *etal.*, 1998, 2001). In addition, and as noted earlier, styrene oxide lung toxicity was completely attenuated in CYP2F2 knockout and CYP2F1 humanized mice, indicating this metabolite is not capable of generating short-term responses in lung indicative of potential lung tumorigenicity.

The objective of this study was to examine the role of CYP2F2 metabolism on the lung toxicity and tumorigenicity for chronic (up to 24 months) exposure to styrene. The design included evaluation of the human relevance of the CYP2F-mediated bioactivation with observations in CYP2F2 knockout and CYP2F1 humanized mice. The test groups were normal C57BL/6 mice (WT, the parental strain for the CYP2F genetically modified mice), and CYP2F2 knockout (KO) and CYP2F1 transgenic (TG) mice on the CYP2F2 knockout genetic background to a high daily (6 h/day, 5 days/week) 120 ppm styrene exposure for up to 2 years. CD-1 mice were included for comparison to the previous chronic study that identified styrene as a mouse lung carcinogen over an exposure range of 20–160 ppm styrene (Cruzan *etal.*, 2001).

## MATERIALS AND METHODS

### 

#### Study Design

Groups of 75 male mice of 4 strains were exposed to either 0- or 120-ppm styrene vapor 6 h/day 5 days/week for up to 104 weeks. The mouse strains included CD-1 (used in Cruzan *etal.*, 2001), C57BL/6 (wild-type for knockout mice, referred to as WT), CYP2F2-knockout (KO), and CYP2F2KO-CYP2F1 transgenic (TG). Male mice were chosen because they generally have a higher incidence of spontaneous lung tumors and appear to be more susceptible to chemically induced lung tumors than females (Cruzan *etal.*, 2001). Five mice per group were removed after 1, 26, 52, and 78 weeks for histopathology, cell proliferation using KI-67, and gene expression analysis (data to be reported elsewhere) of lung tissue. An additional 5 mice per strain per group were terminated at 26 weeks for cell proliferation analysis by BrdU incorporation (data not included due to poor staining). The lungs of all decedent mice and all mice surviving to 104 weeks were examined histopathologically.

#### Animals

About 160 male CD-1 mice were obtained from Charles River, Kingston, NY, at 5–6 weeks of age. About 160 each of C57BL/6, CYP2F2(–/–) (KO), and CYP2F2(–/–) 2F1,2A13, 2B6-transgenic (TG) mice were obtained from Taconic Farms (Taconic Farms Inc, Germantown, NY) at 4–5weeks of age. A total of 150 mice per strain (75 each for control or styrene exposure) were randomly assigned to treatment groups for the study. Descriptions of the generation of the KO (Cruzan *etal.*, 2012; Li *etal.*, 2011) and TG (Cruzan *etal.*, 2013) mice has been previously described. The TG mice also expressed human CYP2A13 and 2B6. They were created by crossbreeding a previously created transgenic strain containing CYP2A13/CYP2B6/CYP2F1 (Wei *etal.*, 2012) with the CYP2F2 KO mice. All mice were acclimated to the laboratory for 14 days before commencement of inhalation exposure. Mice of each strain were stratified by weight and randomly assigned to control or exposed groups. Mice were identified by ear tag and cage card.

Mice were housed individually in polycarbonate cages during nonexposure periods with microisolator lids, Alphi-dri cellulose bedding (Sheppard Products, Kalamazoo, MI), and enrichment products. Mice were fed Certified PicoLab Rodent Diet 20 (Lab Diet, St. Louis) *ad libitum*. Reverse-osmosis treated tap water was supplied *ad libitum* in polycarbonate water bottles with stainless steel sipper tubes.

During exposure periods, mice were housed individually in hanging stainless steel wire-mesh cages (Hazelton M-48) contained within an 8-m^3^ inhalation chamber. Water, as above, was available *ad libitum* via automatic lixits built into the inhalation exposure cage system. No food was present during exposures.

Temperatures were set to maintain 20–26°C, with relative humidity of 30–70%. Lighting was controlled at 12 h on, 12 h off each day. Environmental conditions in the animal rooms and inhalation chambers were monitored continuously.

The animal facility and procedures were accredited by the Association for the Assessment and Accreditation of Laboratory Animal Care International.

#### Inhalation Exposures

Styrene monomer PO-11 Bulk Grade (CAS No. 100-42-5, 99.95% pure) was received approximately quarterly from Lyondell Chemical Company, Houston, TX. A certificate of analysis accompanied each shipment. An inhibitor of styrene polymer formation, t-butyl catechol, was added to the styrene by the producer at 10–15 ppm. Styrene was stored at ambient temperature in an air conditioned room.

Styrene vapor was generated by metering liquid styrene, using an FMI pump, into the upper portion of a J-tube filled with glass beads. Liquid styrene flowed down over the beads. Nitrogen, approximately 25 l/min, flowed upward through the glass beads to carry vapors out of the J-tune. A heating jacket, which did not exceed 145°C, was used to warm the J-tube and contents and aid vaporization. The rate of liquid styrene introduction into the J-tube was regulated to produce 120 ppm (±10%) styrene vapor in the inhalation chamber. A similar set was used for the control chamber, except no styrene was metered into the J-tube.

The nitrogen stream coming from the generating system was mixed with HEPA-filtered air about 5 feet from the inlet to the inhalation chamber. One 8-m^3^ chamber, which housed mice from all 4 mouse strains, received no styrene vapor. These served as the control group for each strain. Another 8-m^3^ chamber, which also housed all 4 mouse strains, received 120 ppm styrene vapor 6 h/day, 5 days/week, except holidays and one day when storms prevented personnel from attendance. Airflow in the chambers was approximately 15 air changes per hour.

In a previous inhalation carcinogenicity study in male CD-1 mice, increased lung tumors were found at 80 and 160 ppm (Cruzan *etal.*, 2001). In a 5-day oral toxicity study, styrene was more toxic to C57BL/6 mice than to CD-1 mice (Cruzan *etal.*, 2012). The 120-ppm exposure was chosen to minimize the possibility of excessive chronic toxicity in the 3 mouse strains based on C57BL/6 mice included in the study, but provide a very high exposure.

Styrene concentration was measured in each chamber using an infrared spectrophometer (MIRAN 1A, Foxboro Co, Norwalk, Connecticut) and was continuously recorded using an automated system. Uniformity of chamber concentration was assured by checking concentration in various locations within the chambers before the start of animal exposures. Chambers were monitored continuously for temperature, humidity, airflow, and differential pressure relative to the room.

#### Toxicity Evaluations

Mice were observed for morbundity twice each weekday and once each week-end day. Moribund mice were sacrificed and necropsied to preserve lung tissue. Mice were weighed within 3 days of arrival, at group allocation, prior to initial exposure, weekly for 13 weeks, monthly until 72 weeks, and weekly thereafter.

Five mice per group were euthanized after 1, 26, 52, and 78 weeks for histopathology and cell proliferation. Mice were euthanized by ip injection of 50 mg/kg Euthasol and exsanguinated from the abdominal aorta. The thoracic cavity was opened and examined for gross lesions. A suture was placed loosely around the trachea. Another suture was placed around the bronchus leading to the right lobes and drawn tight. The right lobes were removed distal to the suture, and stored for genomic analysis. The trachea was cannulated below the larynx and the lung was infused with 10% neutral-buffered-formalin under hydrostatic pressure until the left lobe was filled with fixative. The suture around the trachea was tightened and the cannula removed to tie off the expanded lungs. The lungs were removed from the carcass and placed in formalin for 48 h; then they were placed in 70% ethanol until processed.

Sections of the left lung of mice terminated at 1, 26, 52, and 78 weeks were stained for KI-67. Cells in 5–11 terminal bronchioles were counted for stained and unstained cells. The pathology in and surrounding the terminal bronchioles was the focus of the examination because this location has been identified as the primary site of styrene-induced lung toxicity and tumorigenicity, and also includes the primary location of club (Clara) cells which have been shown to be enriched in CYP2F2 activity in mice (reviewed in Cruzan *etal.*, 2009).

The left lobe of the lung was embedded in paraffin; 5-μm sections were cut, mounted on slides, and stained with hematoxylin and eosin. Tissue was evaluated for pathologic lesions by a board certified pathologist. In some cases, mice died unexpectantly and lung tissue was not of sufficient quality for histopathologic evaluation. The number of lungs examined represents the number of mice whose lungs were evaluated.

#### Statistics

##### Body weight analysis

Body weight data was analyzed using the statistical tests provided by the Provantis software system (version 9.3.1, Instem, Conshohocken, Pennsylvania). A one-way analysis of variance (ANOVA) was used (per time point). If significant, the ANOVA was followed by a Dunnett’s test to compare styrene concentration groups with the control group.

##### Survival analysis

Survival data were analyzed using SAS version 9.2 (SAS Institute, Cary, North Carolina). Survival curves for the control and styrene exposure groups of each mouse strain were estimated separately by the Kaplan and Meier procedure. Animals found dead of other than natural causes were censored from the survival analyses. Styrene-exposed mice were compared with control mice for each mouse strain using the nonparametric log-rank test (*P* < .05).

##### Analysis of lung neoplasms and nonneoplastic lesions

For statistical analysis of lung neoplasms and nonneoplastic lesions, a Fisher’s Exact test was applied where the control group incidence was compared with the styrene concentration group incidence for each mouse strain. A *P* value <0.05 was used as the criterion of statistical significance.

## RESULTS

### 

#### Chamber Concentration

The grand means (± SD) of analytical styrene concentrations for the 104-week exposure group were 0.0 (±0.0) and 120.1 (±1.7) ppm for target exposure concentrations of 0 and 120 ppm, respectively.

#### In-Life Evaluations

There were no signs of styrene-induced toxicity in any of the 4 strains of mice based on general observations of behavior or activity. Mice of all 4 strains exposed to 120 ppm styrene survived longer than their respective controls (Table 1). Survival in control CD-1 and WT mice was not statistically significant from each other. Although KO control mice survived better than the other 3 strains, there was no clear explanation apparent from either the animal observations or histopathology. KO mice exposed to 120 ppm styrene survived approximately the same as KO controls over the entire study and better than styrene-exposed mice of the other strains. No reason was identified. Importantly, however, the results with the various strains indicate that the genetic cytochrome P450 manipulations in these mice did not compromise overall long-term survival of these mice relative to their WT founder strain or to CD-1 mice.

**Table 1:** Survival Through 104 Weeks of Exposure to 0- or 120-ppm Styrene

Strain	CD-1		WT		KO		TG		
Exposure Conc. (ppm)	0	120	0	120	0	120	0		120
Initial No.	50	50	50	50	50	50	50	50	
Week 68	45	42	43	50	46	49	41	49	
75	43	40	39	47	46	49	38	49	
91	21	22	19	32	36	35	22	26	
95	18	18	15	28	30	33	19	21	
104	7	12	4	18*	21	23	9	14	

* Statistically longer life expectancy, compared with WT controls, *P* < .001.

CD-1 mice exposed to 120 ppm styrene weighed less (2–13%) than the CD-1 controls (Table 2) from week 1 through week 96. WT mice exposed to 120 ppm styrene weighed less (2–10%) than WT controls from week 1 through week 95. KO mice exposed to styrene weighed less (up to 7%) than KO controls from week 4 through week 93. There were no differences between weights of TG mice exposed to 120 ppm styrene and the TG control mice.

**Table 2:** Mean Body Weights During 104 Weeks of Exposure to 0- or 120-ppm Styrene

Strain	CD-1		WT		KO		TG	
Exp. Conc. (ppm)	0	120	0	120	0	120	0	120
Week 1	31.1	30.4*	24.2	23.6*	23.6	23.6	24.4	24.6
24	46.9	42.4	34.3	31.6*	30.0	29.3*	32.1	32.3
52	51.2	45.3*	40.9	37.2*	34.4	34.4	36.8	36.7
78	49.8	44.0*	41.3	38.7*	35.6	34.3*	37.7	37.4
104	46.7	44.6	38.6	38.7	36.9	35.4	37.4	36.8

* Statistically significantly different from respective control, *P* < .05.

#### Lung Histopathology

Slight degeneration of epithelial cells lining terminal bronchioles characterized by individual epithelial cells with shrunken, sometimes hypereosinophilic cytoplasm, and small, dark nucleus with condensed chromatin was seen in some CD-1 mice exposed to 120 ppm styrene for 1, 26 or up to 104 weeks, compared with CD-1 controls (Table 3). No degeneration was observed in the 3 styrene-exposed CD-1 mice specifically examined at 52 or 78 weeks. The overall incidence of slight degeneration was 17 of 69 mice. Likewise, slight degeneration of epithelial cells lining the terminal bronchioles was seen in some WT mice exposed to 120 ppm styrene for 1, 26, 52, 78 or up to 104 weeks. The overall incidence was 45 of 70 mice.

**Table 3:** Degeneration of Epithelial Cells in Terminal Bronchioles During 104 Weeks of Exposure to 0- or 120-ppm Styrene

Strain	CD-1		WT		KO		TG	
Exp. Conc.	0	120	0	120	0	120	0	120
Week 1	0/5	3/5	0/5	5/5	0/5	0/5	0/5	0/5
26	0/5	4/5	0/5	4/5	0/5	0/5	0/5	0/5
52	0/5	0/3	0/5	1/5	0/5	0/5	0/5	0/5
78	0/5	0/3	0/5	1/5	0/5	0/5	0/5	0/5
Up to 104	0/50	10/53	0/50	34/50	0/50	0/50	0/50	0/50

**Figure 1:**
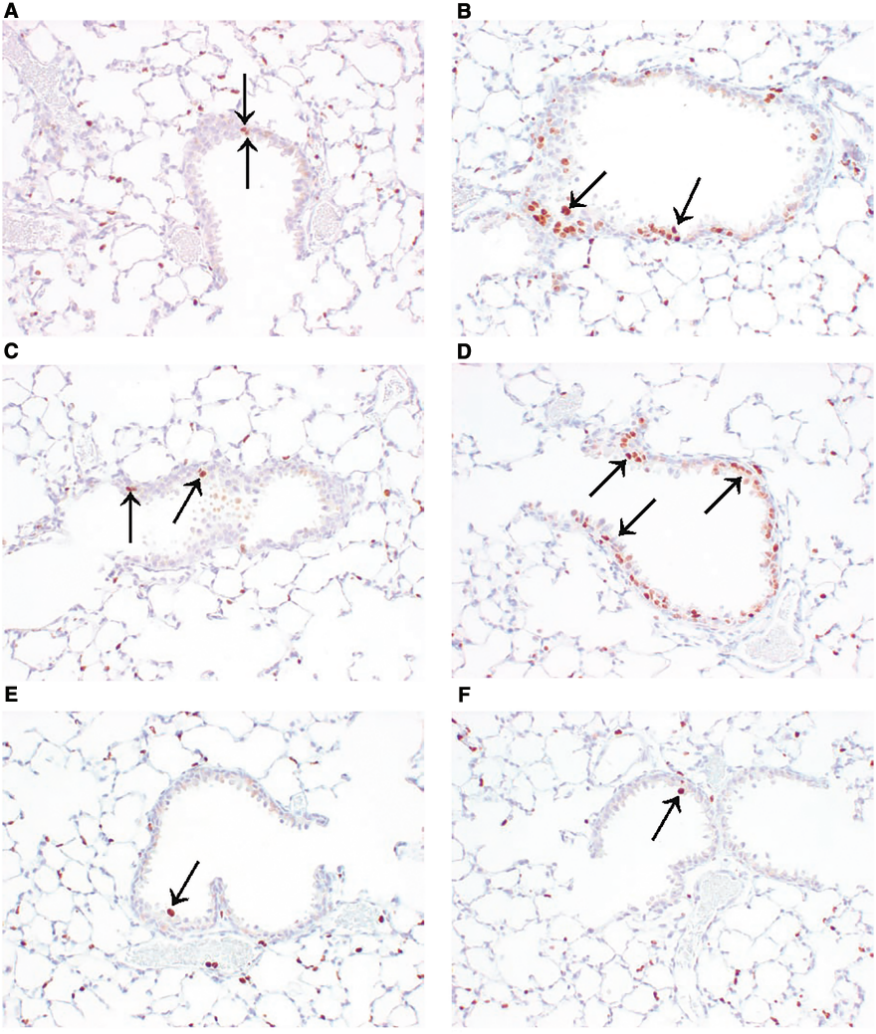
Ki-67 positive stained epithelial cell(s) (arrows) in terminal bronchioles. 20×. A, Animal No. 103. CD-1 0 ppm styrene. B, Animal No. 203. CD-1 120 ppm styrene. C,Animal No. 301. WT 0 ppm styrene. D, Animal No. 403. WT 120 ppm styrene. E, Animal No. 601. KO 120 ppm styrene. F, Animal No. 805. TG 120 ppm styrene.

Four- to five-fold increased cell replication occurred in terminal bronchioles of CD-1 and WT mice exposed to 120 ppm styrene for 1 week compared with CD-1 and WT controls (Table 4 and Figure 1). At weeks 26, 52, and 78, there was no increase in cell replication in CD-1 and WT mice. There was no increase in cell replication in KO or TG mice at any time point.

**Table 4:** Cell Proliferation (% KI-67 labeled cells) in Terminal Bronchioles During 78 Weeks of Exposure to 0- or 120-ppm Styrene

Strain	CD-1		WT		KO		TG	
Exp. Conc.	0	120	0	120	0	120	0	120
Week 1	5.3	20.9*	5.5	31.1*	4.5	3.0	5.1	3.7
26	3.6	2.9	5.6	4.3	4.0	4.9	6.7	4.7
52	0.7	2.1	1.9	1.1	0.8	0.7	0.5	0.4
78	0.6	0.4	1.3	0.7	0.9	0.8	0.8	0.6

* Statistically significantly different from respective control, *P* < .05.

Proliferative changes were found in CD-1 and WT mice exposed to 120 ppm. These consisted of hyperplasia in the terminal bronchioles characterized by increased numbers of unevenly sized epithelial cells “piling” up in multicellular layers, sometimes extending into the alveolar ducts (Table 5) and tumors (Table 6).

**Table 5:** Hyperplasia in Terminal Bronchioles During 104 Weeks of Exposure to 0- or 120-ppm Styrene

Strain	CD-1		WT		KO		TG	
Exp. Conc.	0	120	0	120	0	120	0	120
Week 1	0/5	2/5	0/5	5/5	0/5	0/5	0/5	0/5
26	0/5	5/5	0/5	4/5	0/5	0/5	0/5	0/4
52	0/4	0/3	0/5	3/5	0/5	0/5	0/5	0/5
78	0/5	3/3	0/2	4/5	0/5	0/5	0/5	0/3
Up to 104	0/48	40/51*	0/52	39/50*	0/49	0/49	0/49	0/51
total	0/67	50/67	0/69	55/70	0/69	0/69	0/69	0/68

* Statistically significantly different from respective control, *P* < .05. At week 78, only 2 WT control and 3 TG treated mice were terminated to preserve as many mice as possible for continued treatment. In other cases where the number examined is less than the number initiated, the lung tissue was not acceptable for examination due to autolysis.

**Table 6:** Total Proliferative Lesions in Lungs Following Exposure to 0- or 120-ppm Styrene for up to 104 Weeks^*a*^

Strain	CD-1		WT		KO		TG	
Exposure Concentration	0	120	0	120	0	120	0	120
No. of mice examined	67	67	69	70	69	69	69	68
Bronchiolar hyperplasia^*b*^	0	50	0	55	0	0	0	0
Epithelial hyperplasia^*c*^	1	3	1	0	1	0	0	0
Bronchioloalveolar adenoma	15	14	3	1	0	0	2	1
Broncioloalveolar adenocarcinoma	7	17	0	0	2	0	1	0
Total Mice with proliferative alterations^*d*^	21	58	4	55	3	0	3	1

a Number of mice with proliferative alteration.

b Hyperplasia confined to terminal bronchiole, with possible extension into alveolar duct.

c Diffuse hyperplasia involving areas of bronchioles and alveoli.

d Mice were counted only once in the total, even if a mouse had more than one alteration.

**Figure 2:**
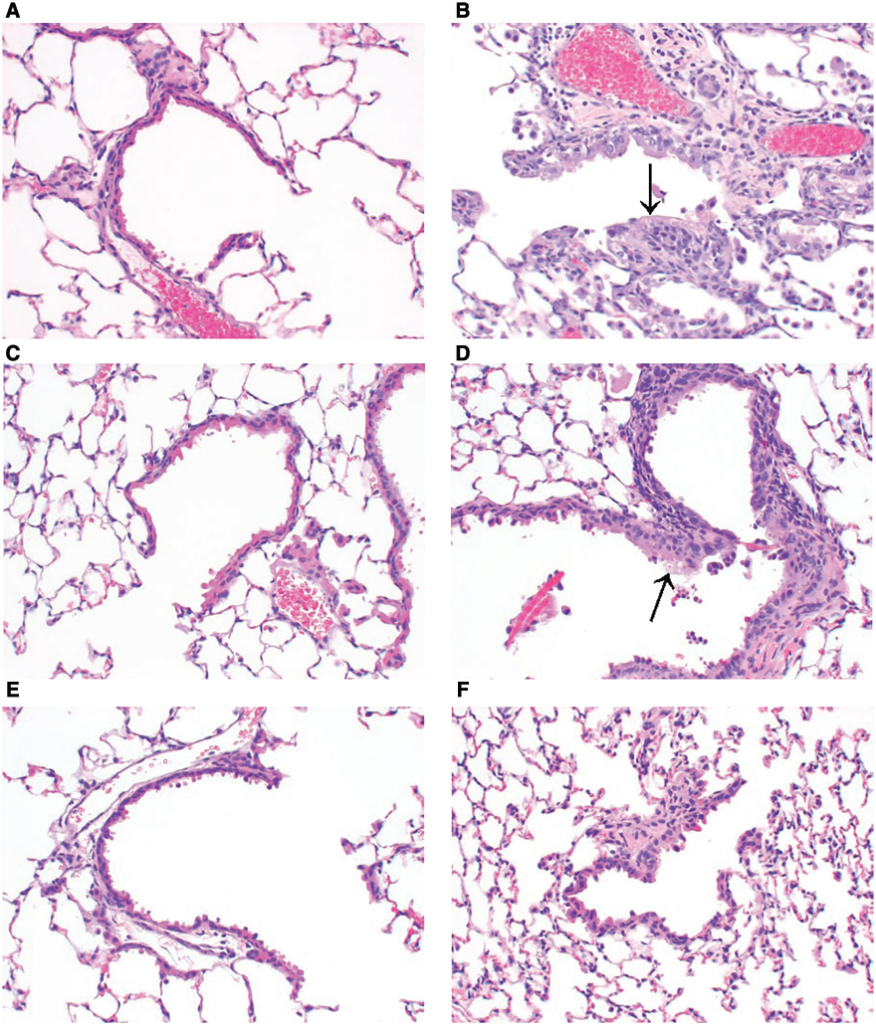
Bronchiolar epithelial hyperplasia. H&E. 20×. A, Animal No. 142. CD-1 0 ppm styrene. B, Animal No. 257. CD-1 120 ppm styrene. C, Animal No. 373. WT 0 ppm styrene. D, Animal No. 438. WT 120 ppm styrene. E, Animal No. 627. KO 120 ppm styrene. F, Animal No. 826. TG 120 ppm styrene.

Hyperplasia was seen in CD-1 mice exposed to 120 ppm styrene vapor at 1, 26, 78 or 104 weeks (Figure 2). Hyperplasia was absent in CD-1 mice exposed for 52 weeks. Overall 50 of 67 CD-1 mice exposed to styrene during this study had hyperplasia in the terminal bronchioles *versus* 0/67 in the CD-1 controls. Similarly, in WT (C57BL/6) mice exposed to styrene, hyperplasia in the terminal bronchioles was found at 1, 26, 52, 78, and 104 weeks. Overall, 55 of 70 WT mice exposed to 120 ppm styrene during the study had hyperplasia in the terminal bronchioles *versus* 0/69 in WT controls. No terminal bronchiole hyperplasia was seen in any of the control or treated KO and TG mice examined up to 104 weeks of exposure. Six mice, including one each in the CD-1, WT, and KO controls, had epithelial hyperplasia (Table 6) that encompassed areas of bronchiolar and/or alveolar tissue, but did not have features of an adenoma. This epithelial hyperplasia was distinguished from the hyperplasia limited to the terminal bronchioles.

Lung adenomas or adenocarcinomas were found in 21 of 67 CD-1 controls and 31 of 67 styrene-exposed CD-1 mice (Table 6); the difference was not statistically significant. C57BL/6 WT mice had a much lower incidence of lung tumors. Increased lung tumors were not found in WT mice exposed to styrene; 3 of 69 controls and 1 of 70 WT-exposed mice had lung tumors. There were no increases in lung tumors in KO (2 were observed in controls) or TG (3 in controls; 1 in treated) mice exposed to styrene.

## DISCUSSION

### 

#### Choosing the Mouse Strains for Evaluating Styrene’s MOA in the Lung

The mouse strains used in this study were developed from C57BL/6 embryonic stem cells (Li *etal.*, 2011; Wei *etal.*, 2012). C57BL/6 mice are resistant to spontaneous lung tumor development (Malkinson, 1989; Miller *etal.*, 2003) relative to B6C3F1 mice (Haseman *etal.*, 1998) or CD-1 mice (this manuscript). However, embryonic stem cells from C57BL/6 are commonly used for developing knockout (KO) mice. A source of embryonic stem cells or procedures for developing KO mice from B6C3F1 or CD-1 mice were not available. Another factor considered in using the C57Bl6 parental strain was a preliminary study (data not published) showing that 200 or 400 mg/kg styrene orally for 5 days caused greater BrdU incorporation in the terminal bronchioles of C57BL/6 mice than in either CD-1 or B6C3F1 mice. Thus, the founder C57BL/6 strain used to produce the KO and humanized TG mice strains was clearly susceptible to early stage styrene lung toxicity equivalent to that seen in CD-1 mice

#### Consistency With Earlier Studies

The incidence of lung tumors was elevated (nonstatistically) in CD-1 mice in this study, an elevation similar to the statistically identified increase in lung tumors in a previous CD-1 mouse chronic inhalation bioassay using doses (20–160 ppm) encompassing the high120 ppm dose used in this study (Cruzan *etal.*, 2001). Higher inhalation exposures are not tolerated in mice (Cruzan *etal.*, 2001). Although the C57BL6 WT mice showed no evidence of increased treatment-related tumorigenicity, they did have early cell proliferation and the later bronchiolar hyperplastic lesions in the current study, paralleling in approximate incidence, time, and location responses in the current and in earlier CD-1 mouse inhalation studies (Cruzan *etal.*, 2001; Green *etal.*, 2001). These styrene induced proliferative lesions in WT mice in this study are precursors of lung tumor outcomes in CD-1 mice, and represent reasonable biomarkers of long-term lung responses in the WT, KO, and TG mice strains.

#### CYP2F2 Metabolism and Lung Tumors

**Figure 3:**
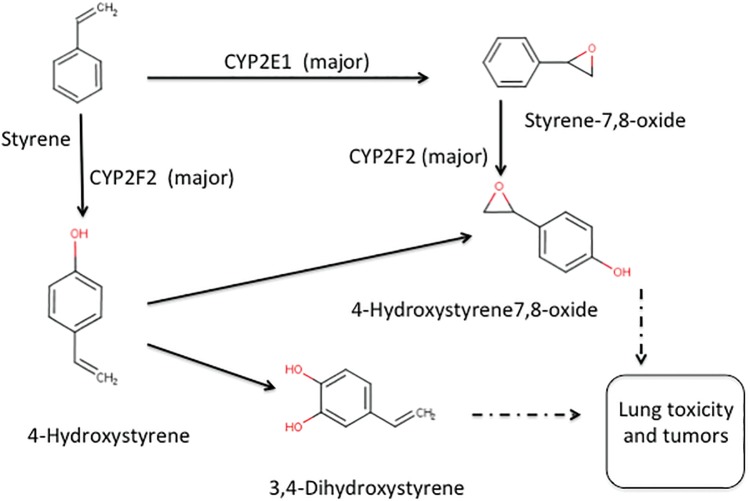
Chemical structures of styrene, styrene oxide, and ring-oxidized metabolites of styrene.

The study was designed specifically to test the hypothesis that CYP2F2 metabolism is essential to styrene-induced proliferative lesions in mouse lungs, by comparing mice with and without CYP2F2 present. The overall findings of this study support the hypothesis that the mouse lung specific chronic toxicity and tumorigenicity of styrene is mediated entirely through mouse-specific CYP2F2 metabolism. Metabolism of styrene by CYP2F2 results in hydroxylation of the aromatic ring. Products include 4-hydroxystyrene, 3,4-dihydroxystyrene, and 4-hydroxystyrene-7,8-oxide (Zhang *etal.*, 2011) (Figure 3). Normal function of CYP2F2 is thought to be hydroxylation of aromatic rings in the synthesis of Coenzyme Q (http://www.informatics.jax.org/go/marker/MGI:88608?header = oxidoreductase; last accessed May 14, 2017). Lungs were examined for cell proliferation and proliferative lesions at 6-month intervals as well as after 24 months, well beyond typical of the short-term studies used in evaluating MoA with virtually all other chemicals. There was no evidence that styrene elicited lung toxicity or tumorigenicity by mode(s) of action other than through CYP2F2 metabolism. In CYP2F2 KO mice, no lung responses were evident, ie, neither tumors nor any degenerative/proliferative lung lesions. The absence of any evidence of histological or cell proliferative toxicity in KO mouse lung at multiple observation times over a 104-week bioassay strengthens the argument that the lung carcinogenic mode(s) of action requires mouse-only CYP2F2 metabolism and other modes-of-action hypotheses are highly unlikely. The CYP2F1 TG humanized mice express metabolically active CYP2F1 (Cruzan *etal.*, 2013). As styrene exposures in these mice did not elicit any treatment-related toxicity or tumors, it is reasonable to conclude that lung tumors in CD-1 or WT mice are not quantitatively, or possibly qualitatively, human relevant.

#### CYP2E1 Metabolism and Pulmonary Responses

Importantly, this study also indicates that that styrene oxide, the primary oxidative metabolite of styrene in both animals and humans (Carlson, 1997; Gadbury *etal.*, 1996; Guengerich *etal.*, 1991), is not associated with styrene carcinogenicity. As CYP2F2 KO mice express equal amounts of CYP2E1 protein in WT and KO mice (Li *etal.*, 2011, 2013), these mice strains are capable of generating styrene oxide. Despite having such activity, styrene lung toxicity was completely attenuated in mice lacking CYP2F enzyme after short-term (Cruzan *etal.*, 2012, 2013) or long-term treatment (this study). In addition, styrene oxide lung toxicity was completely attenuated in CYP2F2 KO mice, indicating that this primary metabolite product of CYP2E1 metabolism is not the proximate lung toxic metabolite of styrene (Cruzan *etal.*, 2012). The lack of a role for CYP2E1 generated styrene oxide in lung toxicity is consistent with the observation that styrene lung toxicity was comparable in both WT and CYP2E1 KO mice (Carlson, 2004). Finally, the absence of toxicity and tumors in rats exposed for 2 years to 1000 ppm styrene (Cruzan *etal.*, 1998) is not consistent with a role of styrene oxide as a key driver of lung toxicity. Blood styrene oxide concentrations measured immediately after termination of a daily nontumorigenic 1000 ppm 6-h inhalation exposure in these rats were 153 and 185 ng/ml for females and males, respectively (Cruzan *etal.*, 1998), whereas concentrations in mice immediately after a daily lung tumorigenic 160 ppm exposure in mice were 20.1 and 35.5 ng/ml for females and males. Despite these substantially higher styrene oxide blood concentrations in rats, no tumors were observed in lung or any other organ/tissue even though blood styrene oxide would be expected to have broad systemic distribution. Tumorigenicity was not observed in 7 other rat studies following styrene exposures conducted by inhalation (2), gavage (4), and drinking water (1) (reviewed in Cruzan *etal.*, 1998). In contrast, styrene was tumorigenic only in lung in various mouse strains treated with styrene by gavage or inhalation (reviewed in Cruzan *etal.*, 2001). These multiple rodent bioassays support a conclusion that styrene, via CYP2E1-mediated styrene oxide formation or ring-oxidized metabolite(s) from CYP2F metabolism, has a very low potential for inducing cancer in any tissue other than mouse lung.

#### Modes of Action Other Than Responses to CYP2F2 Metabolites

Because there were no cell proliferative or histological responses (degeneration, terminal bronchiole or alveolar hyperplasia) in KO mice throughout the entire 104-week study, it is highly unlikely that styrene could cause tumors by alternative mode(s) of action functioning beyond that initiated through CYP2F2 metabolism. This conclusion, that styrene mouse lung toxicity and tumorigenicity is mediated through mouse-specific CYP2F2 metabolism, is reinforced by the lack of responses in CYP2F2 KO and CYP2F1 TG mice following chronic exposure. In these genetically modified mice, possible styrene metabolism by the human CYP2F isoform or that of native CYP2E1 in the CYP2F2-KO mice, also is insufficient to initiate any pre-neoplastic events resulting in potential cancer outcomes.

#### Human Epidemiological Observations of Styrene-Exposed Workers

A large 15 826 worker cohort study of styrene composite workers exposed in multiple production plants between 1948 and 1977 with vital status follow-up through 2008 found no coherent evidence that styrene increased the risk of lung cancer, or for a spectrum of other cancers including lymphatic, hematopoietic or pancreatic tumors (Collins *etal.*, 2013). Although the Standard Mortality Ratio (SMR) for lung tumors was 1.34 (1.23–1.46), additional analyses attributed this increase to smoking due to an inverse relationship between lung cancer deaths and length of exposure, ie, excess lung cancer deaths were noted primarily in shorter term workers. In addition, increased risks of lung cancer were not observed after consideration of increasing cumulative, peak, duration or average exposures. A particular strength of this study was that daily occupational exposures to styrene in this industry and era averaged 30–50 ppm, with peak exposures exceeding 200 ppm; these are in the range of the lung-tumorgenic styrene exposures in mice (20–160 ppm; Cruzan *etal.*, 2001). This study also benefited from its large size and extended follow-up of the study population, providing additional power to more precisely evaluate rare cancers with long latencies. Based on this analysis of a large human cohort, the lack of epidemiological evidence for styrene tumors in humans provides further support of the conclusions of the current study with CD-1, WT, KO, and TG mice: mouse lung tumors and preneoplastic responses seen in CD-1 and WT mice are not human relevant.

#### Summary

The results of this chronic study in CD-1, C57BL/6, and 2 strains of engineered mice on the C57BL/6 background indicate that mouse-specific CYP2F2 metabolism of styrene represents an obligatory key event necessary for the production of downstream biological events ultimately resulting in lung tumors. If this obligatory step is missing (as in the KO mice) or significantly diminished (as in the TG mice), neither lung toxicity nor tumorigenicity takes place. The complete absence of toxicity and/or preneoplastic responses in these KO and TG mice at any stages of this chronic study makes it highly unlikely that there are any other modes of action contributing to styrene-induced mouse lung tumors.

## FUNDING

This study and participation by GC were funded by The Styrene Information and Research Center, Washington, DC.
